# Rheumatoid meningitis in the absence of rheumatoid arthritis: 2 cases

**DOI:** 10.1186/s12883-024-03751-8

**Published:** 2024-07-15

**Authors:** Juan Yang, Lu Liu, Jiahui Peng, Boya Ma, Xiao Yang

**Affiliations:** Department of Neurology, General Hospital of Ningxia Medical University, 804 Shengli Street, Xingqing District, Ningxia, Yinchuan 750000 China

**Keywords:** Rheumatoid arthritis, Rheumatoid meningitis, Unilateral limb weakness

## Abstract

Rheumatoid meningitis (RM) is a rare extra-articular manifestation of rheumatoid arthritis (RA) that has been increasingly recognized by neurologists. However, the diversity of its clinical manifestations makes its diagnosis difficult. RM does not have a unified diagnostic standard, and its link with RA needs to be studied further. Here we report two cases of RM without a history of RA. The first patient, an 80-year-old woman, presented with sudden unilateral limb weakness, with brain MR showing abnormal signals in the leptomeningeal of the right frontal parietal. Subarachnoid hemorrhage was excluded after imaging examination, and infectious meningitis was ruled out after cerebrospinal fluid (CSF) examination. The patient was diagnosed as having RM, she had increased levels of CCP and AKA, the markers of RA, but no history of the disease or other clinical manifestations of it. Another case, a 65-year-old man, was hospitalized with Bell’s palsy. We found that he had intracranial imaging changes highly consistent with those characteristic of RM during his routine examination. Except for the left peripheral facial palsy, the patient had no other neurological signs or symptoms and no RA history. After a careful physical examination, we found no joint or other manifestations or serological abnormalities consistent with RA (RF, CCP, AKA, etc.). However, after excluding infection meningitis and considering the patient’s unique imaging results, we diagnosed him as having RM. We report these two cases as references for clinical diagnosis and treatment of RM, providing a discussion of our rationale.

## Background

Rheumatoid meningitis (RM) is considered an intermediate or advanced stage complication of rheumatoid arthritis (RA). As early as 1967, Ouyang R reported central nervous system involvement secondary to rheumatoid disease [1] in a patient with vomit, speech difficulties, confusion, and seizures during treatment for RA. An autopsy after the patient’s death, revealed diffuse lymphocytic and plasma-cell infiltrates in both temporal lobes, marked arachnoid thickening, and focal granulomatous responses in some areas. There was also vasculitis and perivascular infiltration of lymphocytes and plasma cells, suggesting extensive central nervous system involvement. Later studies demonstrated that the central nervous system damage of RM is mainly manifested on images by meningeal involvement, and the designation of RM was first used in 1983 [2]. In early reports, RM appeared always in typical patients with RA. Those patients were often reported to experience focal weakness, speech disorders, cranial neuropathy, headache, seizures, stroke-like symptoms, psychiatric symptoms, supranuclear paralysis, and parkinsonism during the RA treatment [3–8]. While the typical clinical manifestations of RM are diverse and lack specificity, its imaging characteristics are more unique and have become the most trusted basis for RM diagnosis. In brain MRIs, focal [9, 10] leptomeninges [11, 12] and arachnoid membranes [13] have prominent abnormal signals. The most common enhanced lesions can be found in the vertical plane on the temporal and the fronto-parietal lobes affecting the upper pole of the brain in a symmetrical or unilateral manner [14, 15]. Although the pathophysiology of RM is unclear, it is considered a local manifestation of a systemic inflammatory condition [16].

RM has not been widely recognized in clinical practices due to its low incidence and, in some cases, RM can be virtually asymptomatic and can develop in individuals with apparently quiescent joint disease, which makes its diagnosis difficult. In fact, we wonder whether RM can manifest itself in patients without an RA diagnosis. Could imaging be the absolute standard for RM diagnosis? Herein, we report the cases of 2 patients without a history of RA, both with highly similar brain MRI findings suggesting the presence of RM after exclusion of other diagnoses. We hope these clinical histories will contribute to a better understanding of this unique clinical phenotype.

## Case 1

An 80-year-old female patient with a history of chronic obstructive pulmonary disease (COPD), hypertension, and acute inflammatory demyelinating polyradiculoneuropathy (AIDP) was admitted to our department with the chief complaint of “episodic left limb weakness”, which had occurred twice and lasted for several minutes every time without loss of consciousness or limb twitching. Upon physical examination, the patient was conscious, without cranial nerve abnormalities, and presented mild weakness of the limbs that was considered a sequelae of a past Guillain-Barré syndrome, pathological signs were absent.

**Fig. 1:**
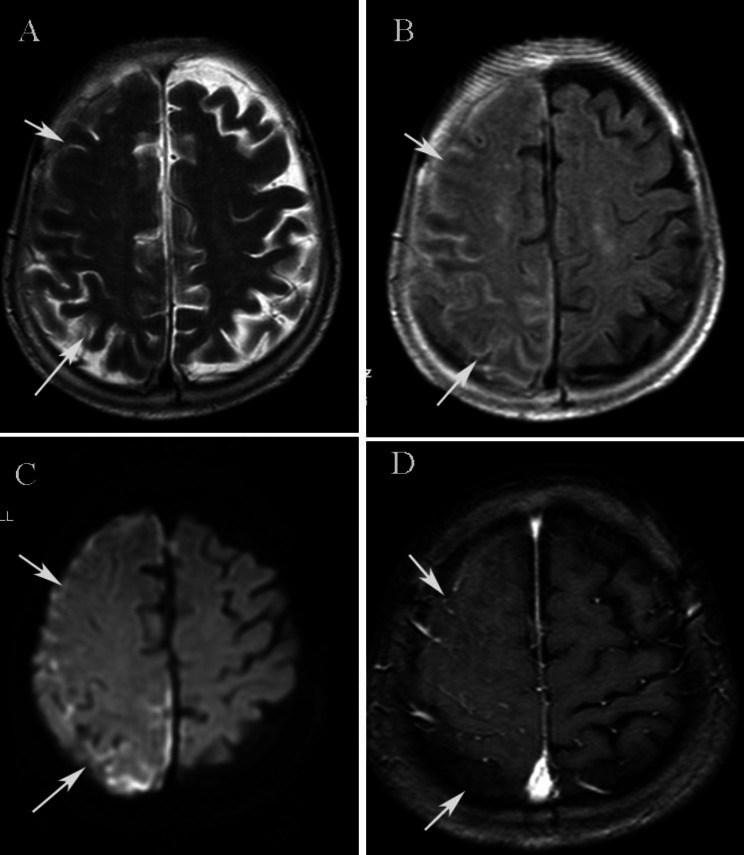
(**A**). T2-weighted MRI showing decreased signal of the right sulci over the right frontoparietal lobe. (**B**). T2-FLAIR demonstrating increased signal with obliteration of sulci due to leptomeningeal thickening over the right frontoparietal lobe. (**C**). DWI showing an increased leptomeningeal signal. (**D**). Axial post-gadolinium T1-weighted image demonstrating unilateral enhancement of the right leptomeninges

Serological tests showed normal routine blood markers; normal serum glucose, liver and kidney function tests, coagulation markers, and urine examination. Tests for tumor markers, levels of folic acid and vitamin B12, and thyroid function tests were all normal. Serological tests for hepatitis B, AIDS, syphilis, and hepatitis C were all negative. Tuberculosis antibody of blood and a PCR assay for Mycobacterium tuberculosis of blood and CSF were negative. Tests for complement components, RF, immunoglobulins, ANA, ENA, and ANCA antibodies were all normal. Serological tests showed a CRP level of 28.5 mg/L (< 10 mg/L), an ESR of 63 mm/h (2–26 mm/h), and a CCP level > 200 RU/mL (0–5 RU/ml), the test for AKA antibodies was positive. The cerebrospinal fluid (CSF) analysis showed a white blood cell count of 15/mm^3^, protein of 0.78 g/L (0.12–0.6 g/L), and glucose of 2.6 mmol/L (2.2–3.9 mmol/L), the CSF bacterial cultures were negative. Brain MRI enhancement showed right parietal leptomeningeal involvement, swelling of gyri, and enhanced meninges (Fig. 1). Abdominal, pelvic and thoracic CTs were normal. The patient did not receive any special treatment during hospitalization, she became asymptomatic and returned home.

## Case 2

**Fig. 2:**
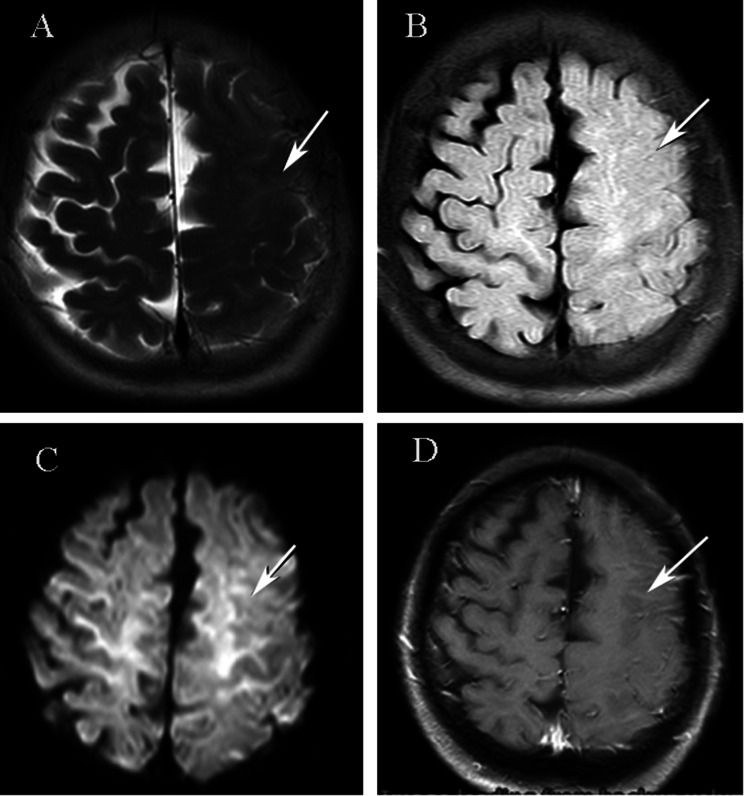
(**A**) T2-weighted MRI showing gyral swelling with disappearance of sulci over the left frontoparietal lobe. (**B**). T2-FLAIR demonstrating increased signal with obliteration of sulci due to leptomeningeal thickening over the left frontoparietal lobe. (**C**). Diffusion-weighted imaging showing an increased signal in the subarachnoid space related to a viscous exudate. (**D**). Axial post-gadolinium T1-weighted image demonstrating unilateral enhancement of the left leptomeninges

A 65-year-old man without a history or manifestations of RA presented with “water leakage and inability to close the left eye completely for 3 days”, accompanied by left-side headache (described as a pinprick pain) without fever, dizziness, nausea or vomiting, with a normal sense of smell, and vision, and absence of limb numbness or weakness. Physical examination was normal except for a left peripheral facial paralysis, otherwise the brain MRI showed abnormal leptomeningeal signal in the left frontoparietal lobes (Fig. 2). Serological routine tests, including those for blood cell counts, blood glucose, liver and kidney function, and coagulation markers, as well as urine examination were all normal. Blood tests for rheumatological conditions were unremarkable. CSF analysis demonstrated normal white cells, glucose, and proteins. The infectious workup was unremarkable and included CSF gram stain and culture. The EMG examination indicated a conduction disorder of the left facial nerve, and the CT examination of the left mastoid bone showed no obvious abnormalities. The patient was given prednisone (40 mg/day orally for 10 days) to treat Bell’s palsy and the left facial palsy symptoms improved significantly after one month. The patient was followed up for 15 more months, and he did not develop any joint or new nervous system symptoms.

## Discussion

The discovery and recognition of RM as a manifestation in some patients with RA has led to a gradual increase in the number of related case reports; however, the pathogenesis remains unclear due in part to the low incidence rate of the condition. RM has been reported in 40–83% of patients with RA [15, 17, 18], more often during middle or late stages of the disease [15]. Because the pathology is mainly limited to focal meningeal and cortical damage, the clinical manifestations include unspecific symptoms of focal deficiency or irritation. Taking all this into consideration, when we admitted the first patient for paroxysmal left limb weakness, our first suspicion was that she was showing symptoms of a transient ischemic attack. However, after assessing the MRI findings showing only right frontoparietal leptomeningeal lesions and ruling out common findings of atherosclerosis and stenosis of the brain arteries or amyloid attacks with subarachnoid hemorrhages, we decided to expand the scope of examinations and found increased serum CCP and AKA antibodies. An analysis of 15 studies by Wang Xueping et al. [19] suggested that the specificity of AKA for RA diagnoses is as high as 94%. In addition, anti-CCP antibody has been suggested to be an early marker of RA with a highly predictive value for its occurrence [20, 21]. In samples obtained more than 1.5 years before the onset of RA, the prevalence of anti-CCP antibody was shown to be approximately 30% [22]. Therefore, we believe that our patient is likely to develop other overt RA symptoms. After a literature review, we discovered that our patient’s unique MRI findings align perfectly with those commonly observed in patients with RM. Therefore, after further excluding infectious and cancerous meningitis, we diagnosed our patient as having RM.

RM used to be a diagnosis with a poor prognosis [23], but recent reviews have highlighted the favorable prognosis of the condition [17, 24]; the mortality rate used to be 81.3% before 1990, and it has been decreased to 3.9% since then. All RM cases reported in the literature show improvement after immunotherapy [17, 24]. RM treatments consist mainly of corticosteroids, more than half of the patients undergo complete recovery, and 14.16% (17/120) of the reported cases have fatal outcomes [15]. Most reported patients recovering completely have received corticosteroids [18]. However, we did not prescribe corticosteroids to our first patient because her symptoms improved on their own after admission. After being discharged from the hospital, the patient’s symptoms did not recur during the follow-up period lasting 1 year and 2 months, and she did not develop rheumatoid-related joint symptoms.

Our first patient had some RA markers and could be considered a patient in a pre-rheumatoid arthritis stage; however, the only evidence for RA we found in our second patient was the RM itself. Our second patient presented no RA-related history or joint lesions, nor did he have any positive RA markers in his blood. The left frontal leptomeningeal abnormalities discovered in the second patient were an incidental finding after a routine MRI, but the patient had no neurological abnormalities that we could ascribe to this lesion. His facial nerve palsy was demonstrably a peripheral injury unexplained by the observed intracranial lesion. During the following 15 months, the patient did not develop any neurological abnormalities or arthralgia. The case of this patient serves as a reminder of the nature of RM, a condition that can appear earlier than RA. A problem arises then, because the diagnosis of RM needs to be based on the presence of RA. How can patients with early RM be diagnosed when they do not present any RA signs or symptoms? Milena Rodriguez Alvarez et al. reviewed 31 cases of RM without a history of RA [24], they did not detect any evidence of active or latent infection in patients with RM and previous RA. In the 6 patients with RM reported by Guan et al. [25], the symptoms of meningitis occurred after the onset of arthritis in five patients and before its onset in one. Taken all this into consideration, we are left to wonder whether RM is not an intrinsic phenotype of RA. Research is needed before being able to clearly define RM.

In conclusion, the histories of our two patients seem to support the evidence for the existence of RM as a separate condition that can appear with mild or no symptoms and in the absence of RA. The RM pathogenesis needs to be clarified by first collecting more patients’ findings.

## Data Availability

No datasets were generated or analysed during the current study.

## Abbreviations

RM: Rheumatoid meningitis
RA: rheumatoid arthritis
CSF: cerebrospinal fluid
RF: rheumatoid factors
COPD: chronic obstructive pulmonary disease
AIDP: acute inflammatory demyelinating polyradiculoneuropathy
